# Vertebra-Level Completeness Analysis in Thoracolumbar Ultrasound Using a YOLO-Based Detection Framework

**DOI:** 10.3390/s26072101

**Published:** 2026-03-27

**Authors:** Sumartini Dana, Chen Zhang, Yongping Zheng, Sai Ho Ling

**Affiliations:** 1 School of Electrical and Data Engineering, University of Technology Sydney, Sydney, NSW 2007, Australia; chen.zhang-6@student.uts.edu.au; 2 Department of Biomedical Engineering, The Hong Kong Polytechnic University, Hong Kong SAR, China; yongping.zheng@polyu.edu.hk

**Keywords:** ultrasound scoliosis, YOLO, vertebra detection, anatomical completeness, vertebra presence matrix, missing vertebrae, vertebra-centric crops

## Abstract

Ultrasound enables radiation-free longitudinal monitoring of scoliosis, but rib shadowing and speckle noise often obscure vertebral structures. Current deep-learning methods present results in terms of localisation accuracy, without directly measuring anatomical completeness. We introduce a vertebra-level completeness model that includes a YOLO-based detection framework and an explicit representation of completeness, the Vertebra Presence Matrix (VPM). The VPM provides visibility into detections across 17 ordinal vertebral levels (T1–T12, L1–L5), allowing us to measure completeness across anatomy rather than just detections. Thoracolumbar ultrasound scans were annotated and divided into train/test sets using a patient-wise split to avoid data leakage. Four model variants were evaluated, including full-spine and vertebra-centric crop representations with single-class and 17-class detection heads. The full-spine detector was less stable in regions of high anatomical variability, such as the upper thoracic and lower lumbar spine. Crops of individual vertebrae were more stable under partial fields of view. The 17-class crop model achieved an mAP50 of 0.929 and a scan-level completeness score of 0.74 using the VPM. These results demonstrate that vertebral completeness can be explicitly quantified and integrated with localisation-based metrics for completeness-aware automated scoliosis evaluation.

## 1. Introduction

Scoliosis is a three-dimensional spinal deformity that necessitates accurate and reproducible assessment of vertebral shapes. While radiographs are the gold standard in clinical practice, they subject patients to cumulative ionising radiation doses throughout follow-up, especially in growing adolescents [1,2,3]. Ultrasound has therefore emerged as a radiation-free modality increasingly adopted for routine monitoring and point-of-care screening [4,5,6,7,8,9].

However, spinal ultrasound acquisition remains technically challenging. The vertebrae are small, frequently obscured by rib shadowing, and subject to significant operator-dependent variability [10]. Thoracic and lumbar sonoanatomy differ substantially [10]. Consequently, many scans contain missing or unclear vertebral levels, particularly in the upper and mid-thoracic spine [11,12]. Recent deep-learning approaches address vertebra localisation and landmark detection [13,14,15]

Missing vertebrae introduce errors that propagate through automated scoliosis analysis pipelines, including curvature estimation, midline extraction, and Ultrasound Curve Angle (UCA) computation [7,16,17]. Vertebrae visibility itself is known to have prognostic value [11]; however, anatomical completeness as a vertebra-level concept remains under-investigated in existing ultrasound-based scoliosis frameworks [5,18].

In most scoliosis imaging studies, including ultrasound and non-radiographic approaches such as mobile back-surface imaging and bare-back image analysis, missing vertebrae are not explicitly quantified and regional failure patterns are rarely reported [4,5,19,20,21].

Despite substantial progress in vertebra localisation and curvature estimation, the issue of vertebral-level anatomical completeness has not been explicitly addressed as a primary evaluation objective in existing ultrasound-based scoliosis systems. To the best of our knowledge, no ultrasound framework currently exists that

- measures completeness of detected vertebrae per level from T1–L5;
- tracks the pattern of missing vertebrae; and
- allows direct comparison of YOLO detector performance across detector configurations.

To address these gaps, we introduce a concise binary encoding of vertebral visibility from T1–L5, termed the Vertebra Presence Matrix (VPM). When paired with existing object-detection-based vertebra detection approaches [22], the VPM provides an ordered and quantitative framework for vertebra-level anatomical completeness assessment, together with a structured representation of missing-vertebra patterns.

The main contributions of this work are as follows: (1) The introduction of a vertebra-level completeness score for quantifying vertebral visibility [11]; (2) A head-to-head comparison of four YOLO configurations (full-spine vs. crop-based input, 17-class vs. 1-class labelling) evaluated on the same ultrasound dataset—an analysis that, to our knowledge, has not been systematically reported in the scoliosis ultrasound literature; (3) The development of a vertebra-centric dataset pipeline with strict case-level separation; and (4) An exhaustive analysis of vertebral consistency patterns, including systematic blind spots at T7 and L2–L5 [10,11].

## 2. Related Work

### 2.1. Ultrasound-Based Scoliosis Assessment

Radiation-free ultrasound has been increasingly investigated as an alternative modality for scoliosis assessment, particularly for longitudinal monitoring in adolescent populations. Early studies focused on system-level validation and feasibility of three-dimensional ultrasound imaging for spinal curvature estimation [4,8,23]. Subsequent work demonstrated that ultrasound-based systems can achieve clinically acceptable agreement with radiographic Cobb angle measurements under controlled acquisition conditions [5,6,7].

However, these methods primarily focus on vertebra localisation in musculoskeletal ultrasound, with relatively limited emphasis on assessing whether all vertebrae in the clinically relevant T1–L5 sequence are consistently visible [18,24]. As a result, existing ultrasound frameworks lack an objective mechanism to determine whether a scan provides adequate anatomical coverage for reliable downstream analysis.

### 2.2. Deep Learning for Vertebra Detection and Localisation

Technological progress in deep learning now allows precise vertebra localisation and landmark detection in both X-ray and ultrasound imaging. CNN- and Transformer-based architectures have been proposed for vertebral segmentation, landmark identification, and curvature estimation [13,25]. These methods demonstrate strong local accuracy, particularly under favourable imaging conditions.

In parallel, deep learning techniques developed for radiographic scoliosis analysis—including two-stage Cobb angle estimation networks and morphology-aware models—have further advanced automated spinal assessment [15,26,27,28,29]. While these approaches achieve high detection performance, they generally operate under the assumption that all clinically relevant vertebrae are present and correctly detected.

Importantly, none of these methods explicitly evaluate vertebra-level anatomical completeness or track missing vertebrae as a first-class outcome. As highlighted in the broader medical imaging literature, missing or corrupted anatomical structures can substantially degrade downstream model performance [17,30,31]. Nevertheless, vertebral completeness has not yet been formalised as a quantitative evaluation metric in scoliosis ultrasound pipelines, and incomplete anatomical representation is often treated as a secondary artefact rather than an explicit evaluation outcome [17,32].

### 2.3. YOLO-Based Detectors for Musculoskeletal Imaging

YOLO-based object detectors have recently been adopted for musculoskeletal and spinal imaging tasks due to their efficiency and suitability for real-time localisation [33]. These detectors have demonstrated strong bounding-box accuracy across a range of ultrasound and X-ray applications.

Despite their effectiveness, existing YOLO-based studies primarily evaluate detection performance using conventional metrics such as precision, recall, and confidence scores. To date, no systematic comparison has been clearly reported between full-spine and vertebra-centric input representations, or between multi-class (17-class) and single-class detector configurations, in terms of vertebral coverage along the full T1–L5 sequence [18].

Moreover, current YOLO-based pipelines do not provide a structured mechanism to assess whether detected vertebrae form a complete and anatomically consistent spinal sequence, particularly under conditions of partial visibility or corrupted anatomical input [17].

### 2.4. Motivation for Vertebra-Level Completeness Modelling

Across ultrasound-based systems, deep learning localisation models, and YOLO-based detectors, a common limitation emerges: vertebral visibility is treated implicitly rather than quantified explicitly. While prior studies acknowledge regional visibility challenges, no existing framework provides a vertebra-level representation that encodes which anatomical levels are present, missing, or inconsistently detected across the spine.

This gap motivates the introduction of the Vertebra Presence Matrix (VPM), which formalises anatomical completeness as a measurable and comparable outcome at the vertebra level. By encoding vertebral visibility from T1–L5 in an ordered binary representation, the VPM provides a structured framework for analysing missing-level patterns and for evaluating detector reliability beyond conventional localisation accuracy metrics. Unlike object-level detection outputs that generate independent predictions, we explicitly model vertebra-level presence, absence, and inconsistency along the spinal axis using the VPM. This constraint-based formulation enables explicit reasoning about which vertebral levels are missing or unreliable. This directly addresses the absence of explicit completeness modelling at the scan level in previous methods.

## 3. Methods

**Figure 1:**
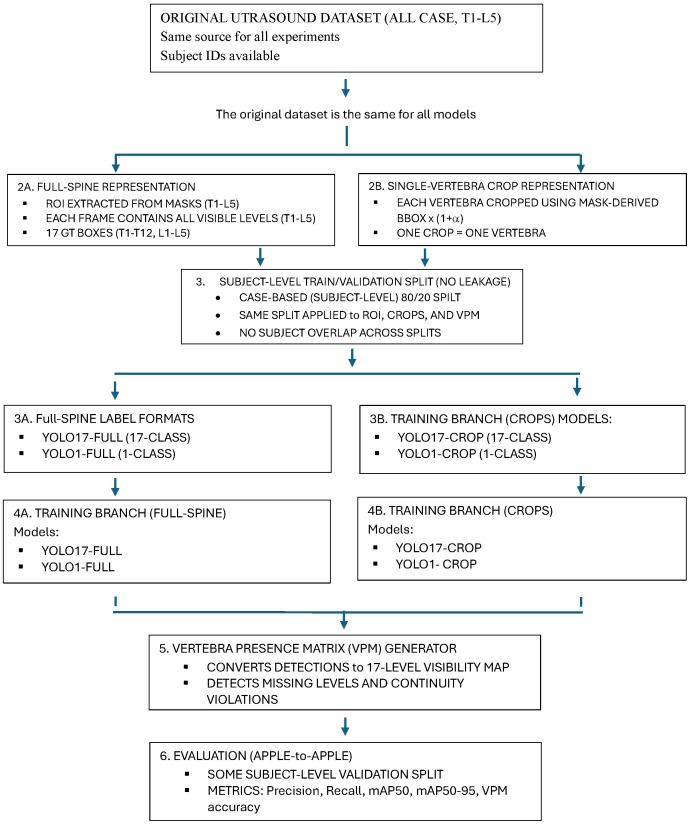
Standardized YOLO experimental pipeline.

This section presents a complete, end-to-end, fully reproducible pipeline for the vertebra-level ultrasound scoliosis analysis task. It is composed of four major modules, namely (i) dataset and annotation, (ii) automatic ROI and vertebra-centric crop generation, (iii) YOLO-based vertebra detection in four controlled configurations, and (iv) Vertebra Presence Matrix (VPM) computation for anatomical completeness evaluation (Figure 1).

The pipeline ensures consistent and fair comparison across input representations (full-spine ROI versus vertebra-centric crops) as well as label granularities (17-class versus 1-class). Subsequent figures detail the VPM-based completeness analysis, including both a representative single-case example and the full ensemble aggregation workflow.

### 3.1. Dataset and Ethics Approval

A retrospectively acquired vertebra-labelled ultrasound dataset from a routine clinical scoliosis screening programme was used. Informed consent was obtained under the aegis of an institutional review board and all ultrasound cine sweeps were fully anonymised prior to analysis.

From each subject, between 10–40 B-mode frames spanning the vertebral levels T1–L5, were acquired using a high-frequency linear transducer (12–14 MHz). Frames affected by excessive rib shadowing, noise, or incomplete visualisation of the vertebral column were excluded.

Vertebral masks were annotated by trained sonographers following a standard anatomical protocol (T1–T12, L1–L5). A second reviewer independently annotated approximately 25% of cases to minimise inter-observer variability. No subjects were recruited for the study. Clinical data available at the time of annotation were used. The annotation dataset is the source for all subsequent ROI, crop, detection, and completeness analyses.

**Figure 2:**
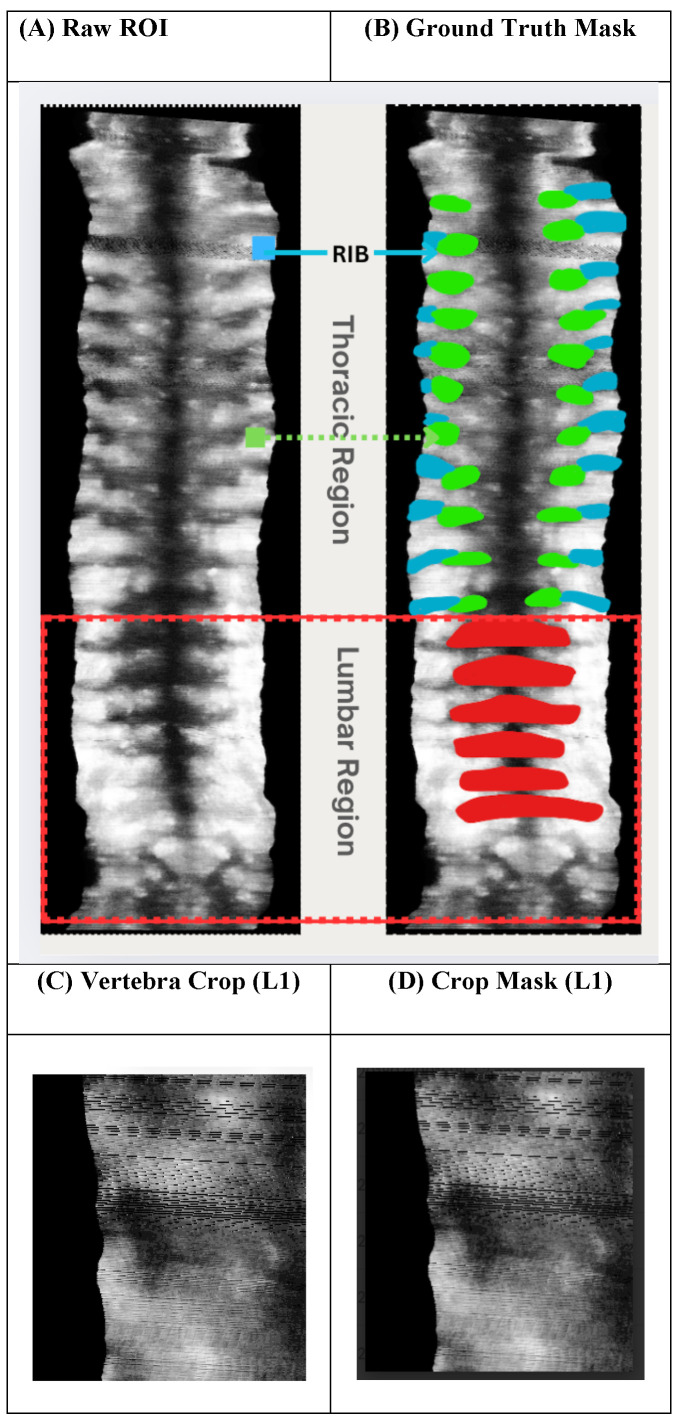
An example of a spinal ultrasound dataset and annotation strategy.

Figure 2 shows representative examples of the ultrasound dataset and the annotation strategy used in this study. The full-spine ROI extracted from cine sweeps includes both thoracic (T1–T12) and lumbar (L1–L5) regions. Thoracic vertebrae are frequently affected by rib structures and acoustic shadowing, leading to reduced contrast and smaller vertebral appearance. In contrast, lumbar vertebrae are larger, more visually distinct, and less influenced by rib artefacts. Figure 2C and Figure 2D illustrate an example vertebra-centric crop and its corresponding mask for a lumbar level (L1), motivating vertebra-dependent crop padding strategies for thoracic and lumbar regions.

### 3.2. Ultrasound Frame Standardisation and Input Preparation

All ultrasound frames were converted to grayscale, normalised to the range [0, 1], and resized to 640 × 640 pixels to standardise the YOLO input across subjects.

The preprocessing pipeline was designed to fulfil three objectives: (i) suppress non-target clutter such as rib artefacts, surrounding soft tissue, and machine annotations; (ii) preserve consistent anatomical framing across cine sweeps; and (iii) enable reproducible ROI and vertebra-centric crop generation for downstream training and evaluation.

Because ultrasound image formation is highly sensitive to artificial edge creation and intensity redistribution, no histogram equalisation, contrast enhancement, or frequency-based filtering was applied in the core pipeline. Similarly, data augmentation techniques known to introduce unrealistic anatomical artefacts such as MixUp, CutMix, Mosaic, and copy paste were explicitly disabled during training to avoid confounding vertebral appearance.

All preprocessing steps, including ROI selection and vertebra-centric crop generation, were executed using deterministic batch-processing scripts with fixed parameters. These steps were applied identically to all subjects without any per-case manual adjustment. The pipeline generates a structured manifest that records the input–output mapping for every processed frame, ensuring full auditability and reproducibility of the preprocessing stage.

### 3.3. Region of Interest (ROI) Definition and Automatic Crop Generation

#### 3.3.1. Automatic Full-Spine ROI Construction

The superior-most visible vertebra (T1) and inferior-most visible vertebra (L5) were determined from the vertebral masks. A rectangular ROI was defined for each frame as the minimum bounding rectangle containing all annotated vertebrae:

(1) $${\mathrm{ROI}}_{i}=\min\mathrm{BBox}\left(T1_{i},L5_{i}\right).$$

This ROI was then applied to all frames from the same subject, preserving anatomical alignment throughout the cine sweep. In this way, the full thoracolumbar column (T1–L5) is consistently present (or purposefully absent), creating a reproducible reference frame upon which anatomical completeness can be assessed.

This definition ensures a subject-invariant and reproducible ROI that can be applied consistently across operators and acquisition conditions.

#### 3.3.2. Automatic Vertebra-Centric Crop Generation

For each annotated vertebra V∈{T1,…,T12,L1,…,L5}, an individual vertebra-centric crop was generated by enlarging its bounding box using a proportional padding factor:

(2) $$\mathrm{Crop}{\left(V\right)}_{i}=\mathrm{BBox}{\left(V\right)}_{i}\times \left(1+\alpha \right),$$

where α=0.15 was selected empirically based on anatomical considerations to provide a conservative balance between retaining sufficient local anatomical detail (e.g., interspinous distances and rib spacing) and avoiding excessive surrounding noise.

Thoracic vertebrae (T1–T12) employed slightly larger padding along the cranio–caudal (longitudinal) direction to preserve adjacent rib-related anatomical cues that are critical for localisation. In contrast, lumbar vertebrae (L1–L5), which are larger and more visually distinct, used symmetric padding.

All ROIs and vertebra-centric crops were generated automatically using a Fully automated pipeline, with no manual correction performed. During training, crops were sampled from ground-truth annotations to enable anatomically aligned crop-based learning. During inference, vertebra-centric crops were extracted from the bounding boxes predicted by the YOLO detector. The padding factor α was fixed for all experiments and was not tuned per subject or per vertebral level.

### 3.4. Dataset Partitioning and Experimental Protocol

A strict case-based 80/20 subject-level split was enforced to prevent information leakage: 160 cases (80%) were used for training, and 40 cases (20%) were reserved for validation. All derived datasets—full-spine ROIs, vertebra-centric crops, and VPM vectors—were partitioned using the same case-level split. No frame, crop, or ROI from the same subject appeared in both splits, eliminating patient-level overlap and preventing memorisation of subject-specific anatomical patterns.

### 3.5. YOLO Training Under Four Configurations

To isolate the effects of input representation and label granularity on detection performance and anatomical completeness, four YOLO-based configurations were trained under otherwise identical, controlled conditions. All models used the same ultrasound dataset, the same subject-level train/validation split, and the same optimisation schedule. The only experimental factors varied were (i) the spatial input representation (full-spine ROI vs. vertebra-centric crops), and (ii) the label formulation (17-class vs. 1-class).

Together, these four configurations enable a direct and controlled comparison of full-spine versus crop-based detection and multi-class versus single-class training.

#### 3.5.1. YOLO Detection Configurations

We consider the following four detector configurations:

1. **YOLO17-Full:** A 17-class detector trained on full-spine ROIs, where all visible vertebral levels (T1–L5) are present in each image, and each vertebra is given a unique class label.
2. **YOLO1-Full:** A single-class detector trained on full-spine ROIs, where all vertebrae are collapsed into a generic “vertebra” class. This eliminates inter-class competition at the cost of losing vertebra-level identity.
3. **YOLO17-Crop:** A 17-class detector trained on vertebra-centric crops, where each input image contains a single vertebra and the corresponding anatomical label (T1–T12, L1–L5).
4. **YOLO1-Crop:** A single-class detector trained on vertebra-centric crops, to serve as an upper bound for the spatial localisation accuracy that can be achieved without enforcing anatomical semantics.

These four configurations enable a direct, apple-to-apples comparison between full-spine and crop-based representations, as well as between multi-class and single-class detection.

#### 3.5.2. Training Protocol

All models were trained using the YOLOv8 architecture with consistent hyperparameter settings across experimental configurations.

Optimisation was performed using the AdamW algorithm with an initial learning rate of $4.8\times 10^{-4}$ and a cosine decay schedule. Training was conducted with a mini-batch size of 16. Early stopping was implemented if validation performance failed to improve for 30 consecutive epochs.

The standard YOLO loss formulation was retained for all configurations, comprising Complete IoU (CIoU) loss for bounding-box regression and binary cross-entropy (BCE) loss for classification.

During inference, non-maximum suppression (NMS) was applied using a confidence threshold of 0.25 and an IoU threshold of 0.7.

For VPM construction, detections were considered valid when the predicted bounding box achieved an IoU ≥0.5 with the corresponding ground-truth annotation.

#### 3.5.3. Implementation Details

All experiments were performed on NVIDIA A100 GPUs available through the UTS International High-Performance Computing (IHPC) cluster. A single, unified Python pipeline was used to train and evaluate all YOLO configurations, ensuring uniformity in implementation and processing.

All datasets (both full-spine ROIs and vertebra-centric crops) were generated programmatically and processed using the same scripts. The software environment, model architecture, optimisation strategy, and evaluation protocol were kept identical across experiments to reduce variability arising from implementation differences.

Figure 1 summarises the complete training and evaluation workflow considered for the YOLO configurations.

### 3.6. Vertebra Presence Matrix (VPM) Computation

**Figure 3:**
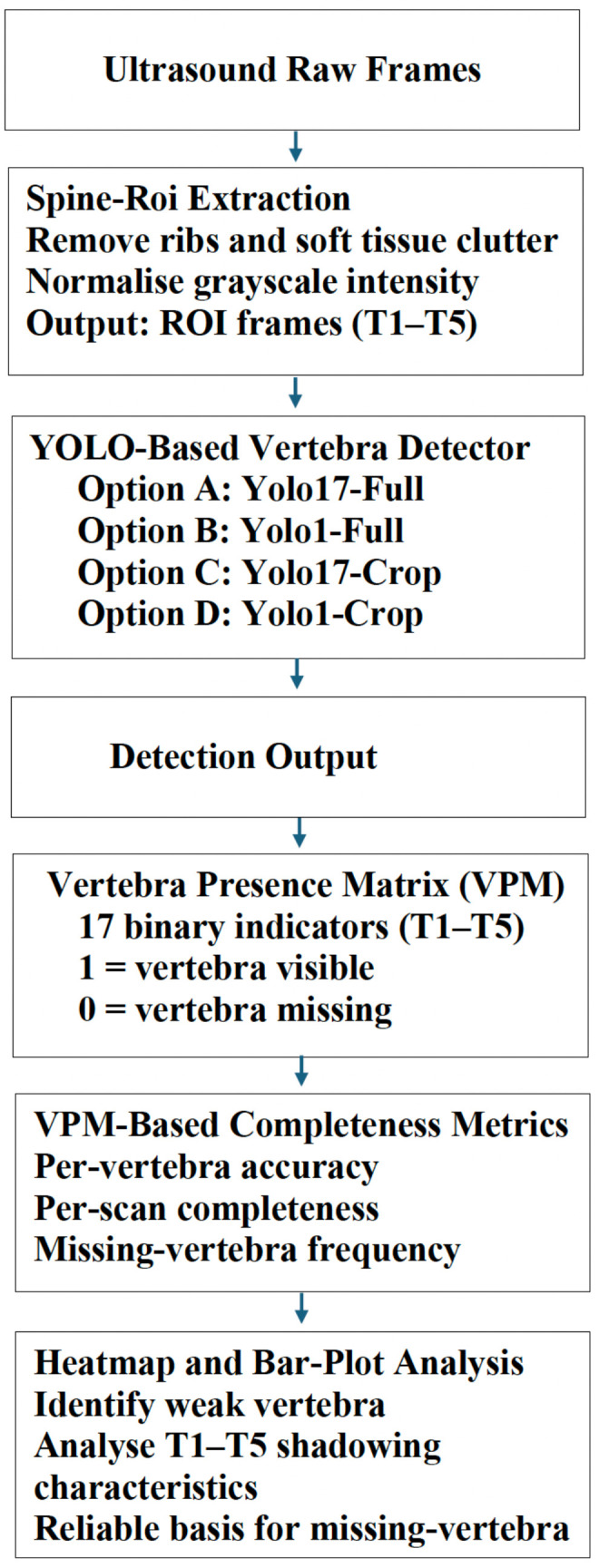
VPM-based pipeline for vertebra-level completeness assessment.

**Figure 4:**
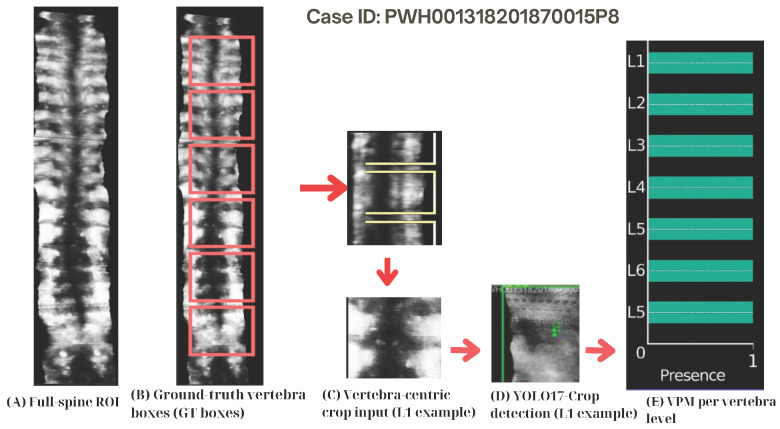
Single-case example of the pipeline from full-spine ROI to Vertebra Presence Matrix (VPM). (**A**) Full-spine ROI. (**B**) Ground-truth vertebra bounding boxes (red). (**C**) Vertebra-centric crop input (L1 example), where yellow boxes indicate cropped regions. (**D**) Detection result using YOLO17-Crop. (**E**) VPM representation indicating vertebra presence (1) or absence (0). Arrows indicate the processing flow.

The Vertebra Presence Matrix (VPM) was introduced to unambiguously and explicitly represent vertebra-level visibility within spinal ultrasound, as well as to allow statistical analyses of anatomical completeness across the thoracolumbar spine. The VPM offers a compact and interpretable representation that encodes which vertebral levels (T1–L5) are visible or missing in each frame and can be easily combined with standard object-detection metrics (which do not encode anatomical completeness by design) to provide vertebra-level visibility labels. Critically, the VPM does not directly affect the YOLO detection process; instead, it is implemented as a post-detection anatomical reasoning layer for completeness assessment. The high-level completeness-assessment pipeline is shown in Figure 3, a representative single-case example is shown in Figure 4.

Raw ultrasound frames are initially pre-processed through the spine ROI extraction stage, which suppresses rib artefacts and soft-tissue clutter and normalises grayscale intensity.

Vertebra detection is then performed using one of the four YOLO configurations described in Section 3.5.1. The resulting bounding-box detections are converted into a binary vertebra-level visibility representation.

The Vertebra Presence Matrix (VPM) encodes vertebra-level visibility for each frame as a binary 17-element vector corresponding to the anatomical vertebral levels (T1–T12, L1–L5):

(3) $$y_{i}=\left[y_{i,1},y_{i,2},\ldots ,y_{i,17}\right]\in {\left\{0,1\right\}}^{17},$$

where $y_{i,v}=1$ indicates that vertebral level *v* is visible in frame *i* according to the ground truth annotation, and $y_{i,v}=0$ otherwise.

#### 3.6.1. Predicted VPM

YOLO predictions are converted into binary visibility indicators ${\hat{y}}_{i,v}$ using a fixed detection-confidence threshold τ:

(4) $${\hat{y}}_{i,v}=\left\{\begin{array}{cc} 1, & \mathrm{if}\;\exists k\;\mathrm{such}\;\mathrm{that}\;s_{i,v}^{\left(k\right)}\geq \tau ,\\ 0, & \mathrm{otherwise}, \end{array}\right.$$

where $s_{i,v}^{\left(k\right)}$ denotes the confidence score of the *k*-th detection associated with vertebral level *v* in frame *i*.

This formulation converts raw YOLO detections into a vertebra-level visibility vector, enabling direct comparison with the corresponding ground-truth VPM representation.

#### 3.6.2. VPM-Based Completeness Metrics

Based on the binary VPM representations, anatomical completeness is quantified using VPM-based accuracy metrics. The overall VPM accuracy is defined as

(5) $${\mathrm{Acc}}_{\mathrm{VPM}}=\frac{1}{NV}\sum_{i=1}^{N}\sum_{v=1}^{V}1\;\left[y_{i,v}={\hat{y}}_{i,v}\right],$$

where *N* denotes the number of frames, V=17 corresponds to vertebral levels T1–T12 and L1–L5, $y_{i,v}$ is the ground-truth visibility indicator, and ${\hat{y}}_{i,v}$ is the predicted visibility.

To analyse level-specific behaviour, per-vertebra completeness is computed as

(6) $${\mathrm{Acc}}_{\mathrm{VPM}}\left(v\right)=\frac{1}{N}\sum_{i=1}^{N}1\;\left[y_{i,v}={\hat{y}}_{i,v}\right].$$

This per-level analysis indicates that there are stable blind zones (i.e., vertebral levels that are consistently missed across subjects), such as T7 or L2–L5, where the human body is complete but the YOLO model does not detect it, quantitatively highlighting anatomical incompleteness that is not reflected in standard object-detection metrics.

### 3.7. How the VPM Enables Reliable YOLO Analysis

YOLO-based detectors generate a collection of bounding-box predictions that encode no explicit information about anatomical completeness or the plausibility of vertebral ordering. To address this limitation, the Vertebra Presence Matrix (VPM) provides a dedicated vertebra-level reasoning layer that enables principled and reliable analysis of YOLO predictions. In particular, the VPM supports the following functions:

1. Anatomical visibility mapping. The VPM translates raw YOLO bounding-box detections into a 17-dimensional semantic visibility signature corresponding to vertebral levels T1–L5. This abstract representation enables consistent comparison of relative vertebral visibility within and across frames and subjects.
2. Detection of missing vertebrae and implausible label sequences. Bounding-box predictions alone do not allow detection of missing vertebrae, false positives, or implausible anatomical orderings (e.g., T3–T6–T4) arising from skip connections or segmentation errors. Vertebral levels T1–T3 and L2–L5, in particular, are frequently missed in clinical ultrasound due to rib cage occlusion and strong acoustic shadowing. The VPM explicitly addresses these failure modes by providing a principled mechanism for identifying missing vertebrae, false detections, and discontinuities with invalid anatomical ordering.
3. Foundation for downstream clinical analysis. The VPM provides a vertebra-level visibility representation that supports downstream scoliosis-related tasks, including apex detection, end-vertebra selection, curvature estimation, and real-time completeness monitoring during clinical scoliosis management workflows.

### 3.8. Statistical Analysis

To evaluate whether vertebral blind zones differ significantly between detector configurations, we performed paired statistical comparisons of per-level detection completeness between full-spine ROI and vertebra-centric crop-based models.

For each vertebral level (v∈{T1,…,T12,L1,…,L5}), completeness was evaluated across frames using the VPM representation, and paired Wilcoxon signed-rank tests were applied to compare completeness scores between configurations.

To control the family-wise error rate across the 17 vertebral-level tests, Bonferroni correction was applied using a significance threshold of α=0.05/17. Vertebral levels with adjusted *p*-values below this threshold were considered statistically significant blind zones.

In addition to statistical significance, we report the Wilcoxon effect size to quantify the magnitude of paired differences:

(7) $$r=\frac{\left|Z\right|}{\sqrt{N}},$$

where *Z* is the normalised Wilcoxon test statistic and *N* denotes the number of paired frame samples.

All statistical analyses were therefore conducted at the vertebral level using paired frame-wise comparisons between detector configurations.

## 4. Results

Results are presented in the following four sections: (i) detection accuracy on all four YOLO configurations; (ii) vertebra-level anatomical completeness, quantified using the proposed Vertebra Presence Matrix (VPM); (iii) sensitivity analysis with respect to vertebra-centric crop scaling; and (iv) the impact of VPM-based anatomical reasoning on correcting implausible vertebral label sequences. Statistical testing was performed to formally assess the presence of consistent vertebral blind zones, as described in Section 3.8.

### 4.1. Detection Performance Across Four YOLO Configurations

**Figure 5:**
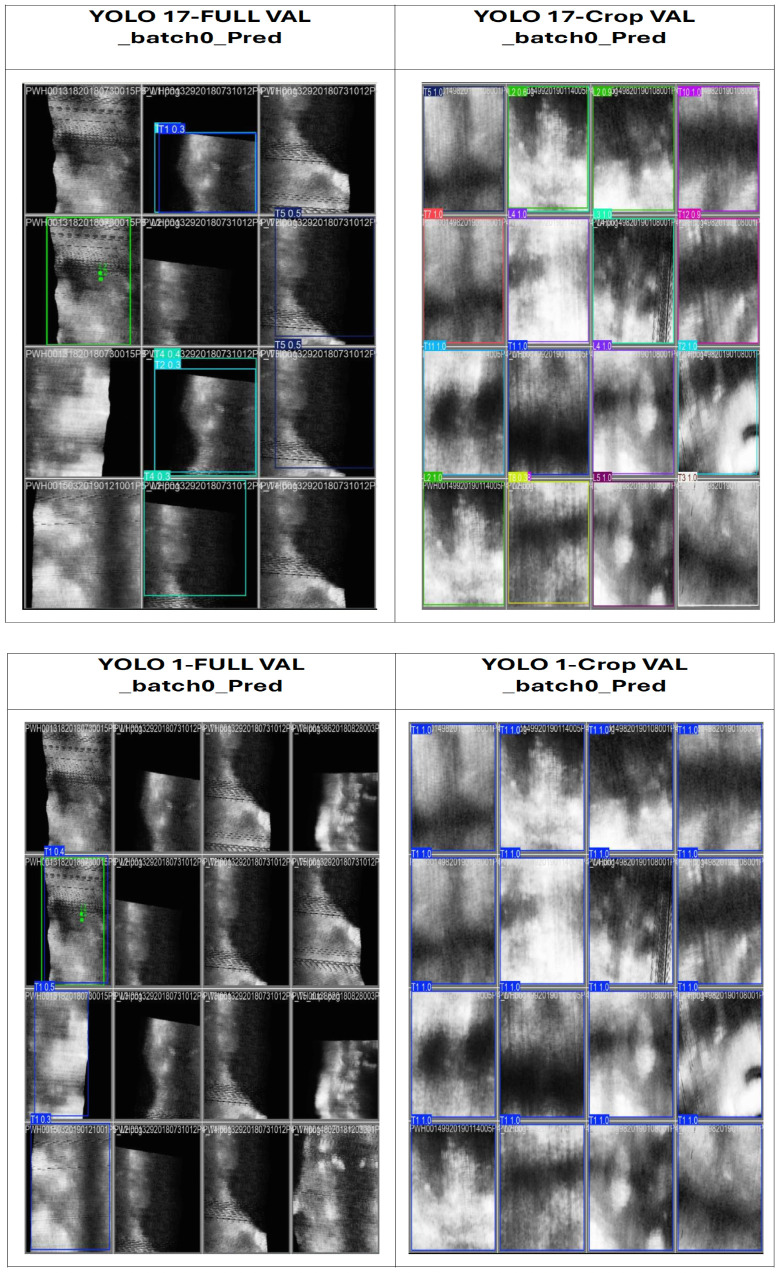
Qualitative comparison of vertebra detection results across the four YOLO configurations.

Detection performance varied substantially across the four YOLO configurations, depending on both the spatial input representation (full-spine ROI versus vertebra-centric crops) and the label formulation (17-class versus single-class). Table 1 summarises quantitative localisation metrics and Figure 5 shows qualitative detection examples.

#### 4.1.1. Full-Spine ROI Detectors

##### YOLO17-Full (17-Class, Full-Spine ROI)

This configuration exhibited limited localisation accuracy and unstable multi-class detection behaviour. As reported in Table 1, YOLO17-Full achieved an mAP_50_ of 0.254 and mAP_50–95_ of 0.176, with precision 0.174 and recall 0.567. Although recall is moderate, the low precision indicates frequent false positives, particularly within rib-shadowed thoracic regions.

Error analysis further showed high false-negative (FN) rates in anatomically challenging regions, including T1–T3 (66–72% FN), T7 (∼45% FN), and L2–L5 (48–63% FN). Bounding-box localisation was also low (IoU =0.21±0.09), suggesting increased inter-class competition among closely spaced vertebral structures within the full-spine field of view.

From an anatomical perspective, full-spine ROI inputs contain multiple overlapping vertebrae, rib-shadow artefacts, and heterogeneous soft-tissue backgrounds. This leads to increased visual ambiguity in upper thoracic regions (T1–T3), the mid-thoracic level (T7), and lower lumbar levels (L2–L5), limiting accurate vertebra-level detections. The coexistence of multiple vertebrae within the same field of view introduces scale variability and inter-class competition, which reduces localisation stability during multi-class training.

##### YOLO1-Full (1-Class, Full-Spine ROI)

Collapsing the multi-class label space into a single generic vertebra class reduced inter-class competition and resulted in improved localisation performance (mAP_50_ = 0.453, mAP_50–95_ = 0.360, precision 0.374, recall 0.618; Table 1). However, this gain was achieved at the expense of anatomical interpretability, as vertebra-level identity was no longer preserved (see Figure 3). Consequently, detections from YOLO1-Full indicate only the presence of spinal structures along the longitudinal axis (T1–L5), without enabling assessment of vertebral completeness or level-specific visibility.

Overall, these findings indicate that full-spine ROI detectors, particularly under multi-class settings, lack the robustness required for vertebra-level analysis due to high miss rates, localisation instability, and limited anatomical interpretability.

#### 4.1.2. Vertebra-Centric Crop Detectors

To address the visibility limitations observed in the full-spine configuration, vertebra-centric cropping was introduced to normalise the spatial context of each vertebra prior to detection. In this approach, each vertebra is evaluated within a localised crop region centred around the annotated vertebral location. This strategy reduces the influence of rib-shadow artefacts, background clutter, and variable field-of-view conditions commonly present in spinal ultrasound images. As a result, vertebra-centric crops provide a more controlled spatial window for detection and allow the model to focus on vertebra-specific features. The following results present the quantitative performance obtained using vertebra-centric crop detectors.

##### YOLO17-Crop (17-Class, Vertebra-Centric Crops)

Vertebra-centric cropping substantially improved detection stability and localisation accuracy. In this configuration, YOLO17-Crop achieved mAP@0.50 of 0.929 and mAP@0.50–0.95 of 0.929, with precision (0.886) and recall (0.868). The nearly identical mAP values suggest stable bounding-box alignment across IoU thresholds under the vertebra-centric crop design. False negatives remained below 12% across vertebral levels, and bounding-box localisation improved (IoU = 0.41 ± 0.07).

These results indicate that evaluating individual vertebrae within a normalised spatial window mitigates rib-shadow artefacts and heterogeneous background interference, enabling consistent vertebra-level detection in the multi-class setting.

##### YOLO1-Crop (1-Class, Vertebra-Centric Crops)

YOLO1-Crop approached the practical upper bound of localisation performance, achieving extremely high detection accuracy (P = 1.00, R = 1.00, mAP@0.50 = 0.995, and mAP@0.50–0.95 = 0.995). Although vertebra-level identity is not preserved in this configuration due to the single-class formulation, the results provide an empirical upper bound for crop-based localisation stability.

Consequently, YOLO1-Crop serves as a reference model for analysing missing-feature behaviour and validating the construction of the Vertebra Presence Matrix (VPM) independently of classification ambiguity.

#### 4.1.3. Key Observations from Table 1

Three consistent trends are observed across the four YOLO configurations (Table 1).

First, input representation strongly influences detection performance. Vertebra-centric crops consistently outperform full-spine ROIs across all localisation metrics, demonstrating that isolating individual vertebrae effectively reduces anatomical clutter and improves detection stability.

Second, label granularity has a pronounced impact on full-spine detection. Collapsing 17 vertebral classes into a single generic class improves localisation performance by reducing inter-class competition; however, this comes at the cost of losing vertebra-level identity, which is essential for downstream anatomical reasoning and completeness assessment.

Third, crop-based inputs enable vertebra-level analysis. Because each crop corresponds to a single vertebra, per-level detection performance becomes measurable and directly supports structured completeness reasoning. This capability is not captured by standard object-detection metrics alone and motivates the introduction of the Vertebra Presence Matrix (VPM) as a complementary anatomical completeness assessment framework.

In full-spine ROI settings, multiple vertebrae may appear within a single image, resulting in ambiguous label assignments and reduced valid detection instances. In contrast, vertebra-centric crops maintain a one-to-one correspondence between images and annotated vertebrae, yielding stable instance counts and reliable per-level evaluation.

In addition to these metrics, localisation accuracy at high IoU thresholds correlates directly with clinical applicability. Tight bounding-boxes imply less ambiguity in vertebral localisation and therefore greater confidence in spinal midline approximation required for Cobb/UCA angle determination. Thus strong performance by crop-based models on mAP@0.50–0.95 suggests alignment consistency.

### 4.2. Vertebra-Level Completeness Patterns (YOLO17-Crop)

Vertebra-centric predictions from YOLO17-Crop are summarised using the Vertebra Presence Matrix (VPM), a post-detection anatomical reasoning layer, enabling T1–L5 vertebra-level completeness analysis (Table 2, Figure 5).

Table 2 presents the VPM-based completeness metrics for the four YOLO configurations, including overall vertebra-level accuracy, per-scan completeness rate, and mean missing-vertebra frequency. Crop-based configurations clearly outperform full-spine ROI detectors in anatomical completeness, reflecting inconsistent vertebral coverage. YOLO17-Crop achieves a mean overall completeness of 0.91 with 0.74 per-scan, indicating consistent vertebra-level coverage while preserving anatomical identity. YOLO1-Crop approaches a practical upper bound (0.98 mean overall 0.94 mean per-scan), highlighting the advantage of vertebra-centric spatial normalisation when label ambiguity is removed. These findings indicate that optimising for mAP alone is insufficient to ensure anatomically complete spinal representation. VPM-based completeness metrics therefore provide clinically relevant additional information for ultrasound scoliosis assessment.

The qualitative comparison of vertebra detection results across four YOLO configurations (YOLO17-Full, YOLO1-Full, YOLO17-Crop, and YOLO1-Crop). Crop-based detectors exhibit more consistent localisation and clearer vertebral boundaries, while full-spine detectors frequently miss vertebrae and produce unstable bounding boxes due to anatomical clutter and rib shadowing.

**Figure 6:**
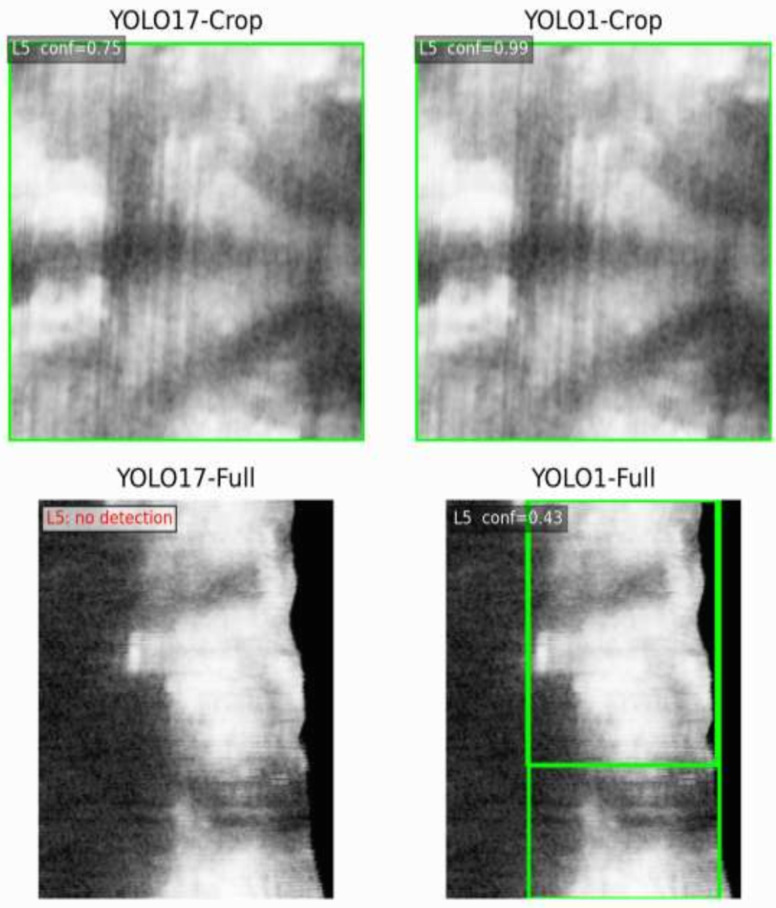
Effect of label granularity on crop-based vertebra detection at level L5.

To validate the effect of label granularity on crop-based vertebra detection, Figure 6 illustrates the predicted bounding boxes produced by YOLO17-Crop and YOLO1-Crop for the same vertebral level (L5) in a representative case. Because both models were evaluated on identical input crops, the single-class model consistently produces higher confidence and more stable localisation, supporting its use as a reliable foundation for VPM construction.

Comparison between YOLO17-Crop and YOLO1-Crop on case PWH001218730015P8 at vertebral level L5. The single-class crop detector yields higher confidence (0.99) and more stable localisation than the 17-class crop detector (0.75), illustrating its suitability as a reference upper bound for VPM construction and missing-vertebra analysis.

T7 exhibited the lowest VPM accuracy (0.729), followed by L2 (0.776) and L5 (0.773), whereas upper thoracic (T1, T8) and junctional vertebrae (T12, L3) achieved consistently higher accuracies (>0.88) (Table 3).

#### 4.2.1. Significant Supported Weak Zone at T7

Our statistical assessment identified T7 as the most consistently low-performing vertebral level. Accuracy at T7 was 0.729 (95% CI, 0.595–0.834), whereas the pooled accuracy across all other levels was 0.841. The reduction at T7 was statistically significant based on a two-proportion z-test (p=0.043).

These findings indicate a reproducible mid-thoracic visibility limitation at T7, likely attributable to rib shadowing and a reduced acoustic window.

#### 4.2.2. Lower (Non-Significant) Completeness Around the Thoraco-Lumbar Transition

We also noted a loss of completeness at the thoracolumbar junction, most pronounced at L2 and L5. In particular, these levels achieved accuracies of 0.776 (CI [0.662, 0.859]) and 0.773 (CI [0.666, 0.854]), respectively. No statistically significant difference was observed between the two conditions (p≈0.11–0.13). It is conceivable that a greater imaging depth as well as disruption by abdominal or pelvic anatomy in the lower lumbar region could deteriorate vertebral contrast, even when vertebra-centric cropping is applied.

#### 4.2.3. High-Completeness Levels and Anatomical Interpretation

**Figure 7:**
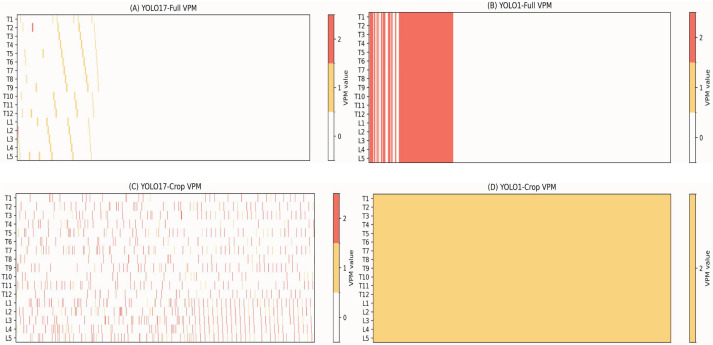
VPM-based completeness heatmaps for four YOLO configurations. (**A**) YOLO17-Full, (**B**) YOLO1-Full, (**C**) YOLO17-Crop, and (**D**) YOLO1-Crop.

On the other hand, vertebral levels more frequently imaged in larger and more stable acoustic windows (i.e., T1 [0.926], T8 [0.913], T12 [0.900], and L3 [0.887]) had a higher completeness. Overall, these findings align with the VPM-based heatmap (Figure 7), confirming a statistically significant weak zone at T7 and a reproducible (though non-significant) drop across lower lumbar levels (L2–L5).

**Figure 8:**
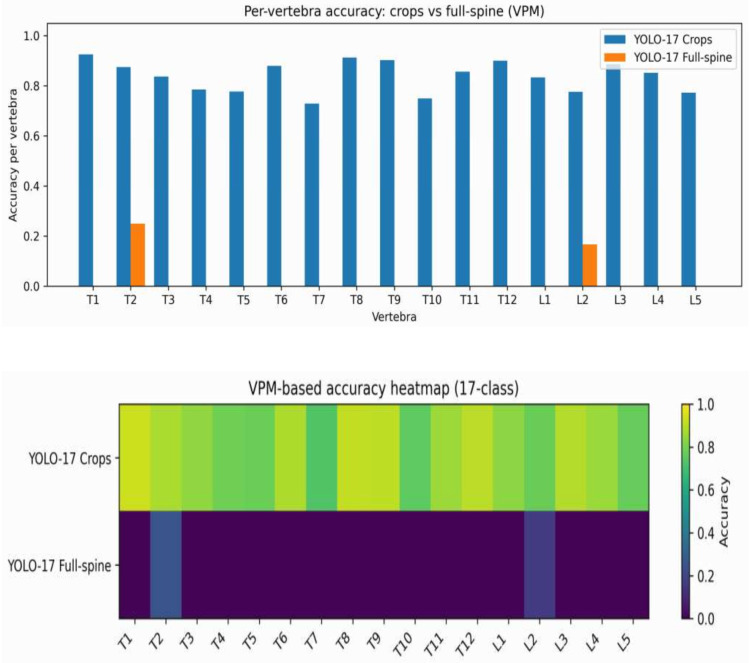
Per-vertebra detection accuracy using VPM-based evaluation.

In summary, we show that the per-vertebra analysis suggests that crop-based detection can consistently monitor anatomical completeness over most vertebral levels, and also provides information about locations with specific visibility problems (Figure 8). This motivates the following robustness analysis (Section 4.3), which evaluates crop scale sensitivity and detection stability under small perturbations.

### 4.3. Robustness Analysis: Effect of Crop Scaling and Detection Stability

To assess the robustness of vertebra-centric detection, we analysed the sensitivity of confidence and completeness to controlled variations in crop scale. This experiment evaluates whether the gains of crop-based detection persist under reasonable changes in anatomical context, rather than depending on a single cropping configuration.

#### 4.3.1. Experimental Setup

**Figure 9:**
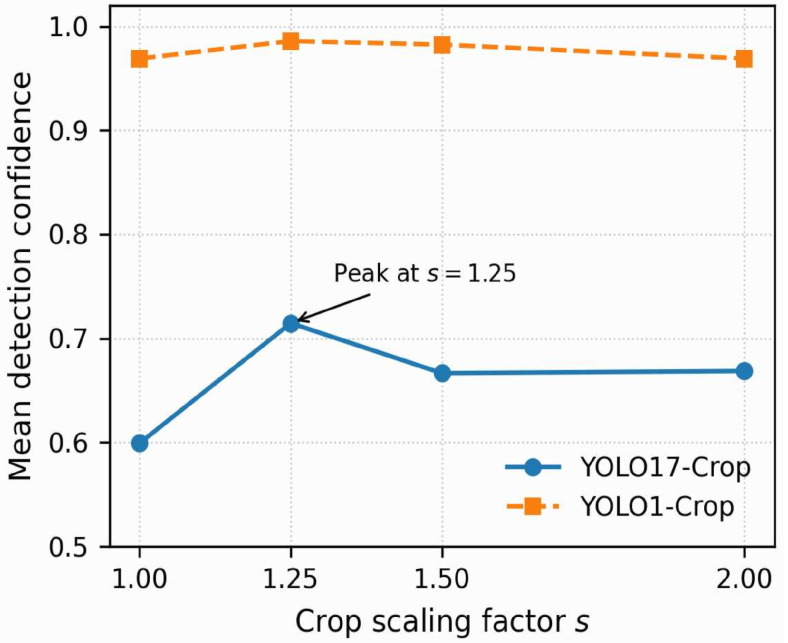
Effect of crop scaling on YOLO detection confidence.

Using the same strict no-leak validation set, vertebra-centric crops were generated with scale factors relative to the original bounding box, s∈{1.00,1.25,1.50,2.00}. Image resolution (640 × 640), preprocessing, and model weights were held constant. For the crop-scaling analysis, inference was repeated at each scale to isolate the effect of spatial context introduced by crop scaling. Detection confidence was computed for each scale setting (Figure 9).

#### 4.3.2. Results and Interpretation

Figure 9 shows that YOLO17-Crop confidence is sensitive to crop scaling, peaking at moderate padding (s=1.25). Tighter crops (s=1.00) reduce confidence, suggesting insufficient anatomical context for robust multi-class discrimination, whereas larger crops (s=1.50–2.00) plateau or slightly decrease, consistent with added clutter from surrounding tissue. In contrast, YOLO1-Crop remains consistently high across all scales, suggesting that the observed sensitivity in the 17-class configuration is primarily linked to label granularity rather than localisation instability. These findings motivate a post-detection anatomical consistency step, evaluated in the next section through the proposed VPM correction.

### 4.4. Effect of VPM Correction on Classification Errors

Despite the localisation performance and confidence reported in Section 4.3, we observed that YOLO17-Crop’s raw class predictions were unstable at the vertebra level. In particular, thoracic vertebrae were often misclassified systematically across all crop scales, even for high-confidence predictions (>0.9). These errors were not confined to noisy detections and did not indicate a failure in localisation. Instead, they reflect an inherent limitation of vertebra classification based solely on single-vertebra crops.

In representative examples (Table 4), vertebra T8 was predicted as T1 with a confidence of 0.99, and T9 was also predicted as T1 with a confidence of 0.95. Similar patterns of high-confidence class collapse were observed at the thoracolumbar boundary (L1–T12) and between structurally similar thoracic levels (T2–T7).

Root-cause analysis of these systematic errors suggests that they arise from fundamental limitations of single-vertebra crop classification:

1. Strong inter-level appearance similarity: Adjacent thoracic vertebrae exhibit highly similar ultrasound morphology, making local appearance insufficiently discriminative.
2. Weak level-specific texture cues: Ultrasound lacks stable, level-specific visual features that uniquely identify neighbouring vertebrae.
3. Loss of global anatomical context: Vertebra-centric cropping discards positional information along the cranio–caudal axis, preventing reliable inference of relative vertebral ordering from local appearance alone.

The proposed VPM addresses these limitations by imposing anatomical sequence constraints on the predicted vertebral order. Rather than retraining or modifying the detector, VPM operates as a post-detection reasoning layer applied to the predicted vertebral sequence to ensure anatomically valid spine-level structure. Specifically, it enforces:

1. Monotonic vertebral ordering (T1 → T12 → L1 → L5);
2. Inference of missing vertebrae through positional and continuity reasoning; and
3. Suppression of spurious or inconsistent outlier predictions using anatomical constraints to eliminate large, implausible level jumps.

After VPM correction, all predicted vertebral sequences were anatomically consistent. When a single vertebra was misclassified with high confidence, the VPM resolved the misclassification without retraining the detector, demonstrating that VPM acts as a complementary global reasoning mechanism rather than a local refinement step.

Overall, these results demonstrate that VPM contributes substantially to ensuring vertebra-level anatomical completeness. While vertebra-centric cropping is a powerful enabler for localisation, global anatomical constraints are necessary to disambiguate vertebral levels and to produce complete, reliable, and clinically meaningful vertebral sequences for scoliosis assessment. Importantly, the VPM does not introduce new detections, but instead constrains existing predictions, preserving detector transparency while improving anatomical validity.

**Table 4:** Representative examples of high-confidence vertebra misclassification corrected by VPM.

Case ID	True Vertebra	YOLO17-Crop Prediction	Confidence	Final VPM Label
PWH0013820180730015P8	T8	T1	0.99	T8
PWH0013820180730015P8	T9	T1	0.95	T9
PWH0013820180730015P8	L1	T12	0.88	L1
PWH0013820180730015P8	T2	T7	0.81	T2
PWH0013820180730015P8	T3	T1	0.91	T3

### 4.5. Inference Speed

Runtime logs from iHPC GPU servers were used to evaluate real-time feasibility in clinical ultrasound workflows. YOLO17 detector averaged around 8 ms/frame, including preprocessing, inference, and postprocessing time. Vertebra-centric cropping and VPM generation added negligible compute time (<2 ms/frame). The complete multi-stage pipeline operates at approximately 10 ms/frame ( 100 FPS), demonstrating real-time capability for online clinical ultrasound screening and monitoring.

### 4.6. Implications for Completeness Monitoring and Downstream UCA Estimation

The proposed VPM-based framework has several direct implications for real-time clinical deployment of ultrasound-based scoliosis assessment.

#### Real-Time Completeness Feedback During Acquisition

Because the VPM is computed along the acquisition sweep (T1–L5), vertebral presence can be explicitly tracked in real time, allowing missing or ambiguously detected levels to be flagged during scanning. This enables operators to adjust probe orientation, pressure, or sweep trajectory to recover missing vertebrae, rather than discovering incomplete coverage after acquisition. This real-time feedback may help to avoid repeated scans and improve inter-operator consistency, which remains a key limitation of current sonographer-assisted systems.

#### Identification of Reproducible Anatomical Blind Zones

Per-vertebra completeness analysis revealed statistically significant and reproducible blind spots, most notably at T7, as well as non-significant but consistent decreases at the thoracolumbar transition (L2–L5). These findings demonstrate that ultrasound visualisation is not homogeneous across the spine. Importantly, this information can be incorporated into acquisition protocols by suggesting alternative probe angles or sweep strategies in regions where rib shadowing and acoustic occlusion are more likely. In this sense, blind-spot detection shifts from post-hoc analysis to an on-the-fly acquisition guide.

#### Improved Apex and End-Vertebra Identification for UCA Estimation

Accurate estimation of the Ultrasound Cobb Angle (UCA) critically depends on the correct identification of apex and end vertebrae. Errors arising from dropped, duplicated, or transposed vertebral detections propagate directly into erroneous angle estimates. Unlike direct angle-regression approaches, the proposed VPM enforces global anatomical consistency by producing an ordered, anatomically coherent vertebral sequence prior to curve estimation. This sequence-level constraint implicitly regularises apex and end-vertebra selection by conditioning the input on a valid spine order.

#### Enabling Ultrasound as a Radiation-Free Alternative for Scoliosis Monitoring

Beyond detection accuracy alone, the proposed framework enhances the clinical trustworthiness of ultrasound imaging as a repeatable, low-risk alternative for paediatric scoliosis follow-up and long-term monitoring. By combining robust vertebra-centric localisation with global anatomical reasoning, the VPM framework addresses two major sources of unreliability in ultrasound imaging: local misclassification and global ordering ambiguity. Both are critical for enabling reliable longitudinal assessment without ionising radiation.

The robustness analysis of crop scaling in Section 4.3 further confirms that anatomical reasoning beyond local appearance cues is required for stable vertebra-level interpretation (Figure 9).

Figure 9 illustrates the trend reported in Table 4: YOLO17-Crop achieves its highest mean detection confidence at an intermediate crop scaling factor (s=1.25), whereas YOLO1-Crop remains stable across all tested scales.

We carried out another sensitivity analysis over crop scaling factors f={1.00,1.25,1.50,2.00} for additional validation of our vertebra-centric crop choice. All optimisation parameters were kept static and only the scaling of the crop was manipulated at inference time, in order to isolate the effects of context-scaling. The plot demonstrates a clear non-monotonic behaviour, where the sweet-spot of detection confidence/localisation robustness was found at f=1.25. Smaller crops led to loss of contextual information, while larger crops re-introduced background clutter and rib-shadow artefacts. This provides empirical justification for choosing α=0.15, as a compromise between context and noise. Crucially, increasing crop size did not wash-out the previously described systematic mid-thoracic misclassification artefact.

Importantly, increasing the crop size did not reduce the previously identified systematic mid-thoracic misclassification patterns. This indicates that the failure mode arises from structural ambiguity rather than insufficient local context. Consequently, enlarging the receptive field through larger crop sizes is unlikely to mitigate these errors. Instead, this finding further motivates the need for global anatomical reasoning via the proposed Vertebra Presence Matrix (VPM). These observations naturally lead into the broader discussion regarding the limitations of local vertebra-centric detection and the need for global anatomical reasoning, as discussed below.

## 5. Discussion

### 5.1. Interpretation of Detection Behaviour

Building upon the sensitivity analysis presented in Section 4.3, detection quality across the four configurations is primarily determined by the input representation. Full-spine ROIs contain multiple overlapping vertebrae, rib shadows and heterogeneous soft tissue, which increases false negatives and fragmented proposals and yields markedly lower mAP than vertebra-centric crops (Table 1). By contrast, crop-based detection isolates a single vertebra at a consistent spatial scale, reducing background competition and improving localisation reliability.

However, high localisation performance does not guarantee correct vertebra-level identity in the 17-class crop setting. Adjacent thoracic levels exhibit highly similar ultrasound texture patterns, and cropping removes global cranio–caudal context, making level identification inherently ambiguous from local appearance alone. This explains the observed high-confidence mislabelling events prior to sequence correction (Table 4).

### 5.2. Blind-Zone Significance and Imaging Limitations

VPM heatmaps (Figure 7) reveal reproducible anatomical “blind zones”, with the most prominent loss around T7 and smaller losses near the thoraco–lumbar transition (L2–L5).

To further characterise this observation, we analysed the VPM validation outputs. In the YOLO17-Crop configuration, T7 failed in 21 out of 28 visible cases. Similar patterns were observed in the lower lumbar region (L2: 27/28; L3: 21/26; L4: 19/27; L5: 26/30). These results suggest that detection sensitivity is consistently reduced in these anatomically challenging regions.

These trends persist even under higher crop scales (Section 4.3), implying that they reflect imaging-related constraints (i.e., rib-shadow occlusion, narrower acoustic windows, and depth-related attenuation) rather than detector instability alone.

In addition to minimising anatomical clutter, vertebra-centred cropping reduces the effective receptive field of the detector head and standardises object scale across samples. This decreases variation in contextual background cues and enables the network to learn robust local morphological patterns specific to vertebrae. Ultrasound images commonly include rib shadowing and acoustic artefacts that produce noisy patterns. Truncating the spatial extent of the ROI further minimises false activations caused by these confounding structures.

Importantly, the VPM in our framework is intentionally formulated as an auxiliary post-object-detection anatomical reasoning layer rather than a modification of the detector architecture itself. This design preserves modularity, interpretability, and compatibility with standard object-detection pipelines, while enabling explicit reasoning about vertebra-level completeness. Enforcing attention priors or temporal consistency directly within detection may further reduce blind-zone effects, but such architectural modifications fall outside the scope of the present study. Here, we intentionally maintain a model-agnostic completeness formulation.

### 5.3. Clinical Implications for Real-Time Ultrasound Use

The proposed VPM framework enables vertebra-level completeness feedback during acquisition: as the probe traverses the spine, detected levels (T1–L5) can be updated in real time, allowing the operator to adjust probe orientation, pressure, or intercostal angle to recover missed regions. Automatic identification of consistently missing levels can further guide targeted re-scanning in challenging zones (e.g., around T7), improving reproducibility and reducing the risk of propagating missing anatomy into downstream curvature estimation (UCA).

### 5.4. Relation to Prior Work

Most prior ultrasound scoliosis pipelines report detection or segmentation accuracy but do not explicitly quantify anatomical completeness along the full vertebral chain. Our results show that standard detection metrics can overstate clinical reliability when systematic missing-level failures occur. By introducing VPM-based completeness measures and linking them to reproducible blind zones, we provide an evaluation layer that is directly relevant to vertebra ordering, apex/end-vertebra selection, and downstream UCA stability.

## 6. Conclusions

### 6.1. Summary of Findings

This study introduces a vertebra-level anatomical completeness formulation for ultrasound-based scoliosis assessment by combining vertebra-centric YOLO detection with global sequence reasoning via the Vertebra Presence Matrix (VPM). Crop-based detection improves localisation robustness compared with full-spine ROIs. However, vertebra-level identity cannot be reliably inferred from local appearance alone in the 17-class setting due to repetitive anatomical patterns. These limitations are addressed by constraining predictions to an anatomically plausible vertebral sequence and explicitly encoding presence or absence at each level. As a result, VPM suppresses recurrent high-confidence mislabelling and enables systematic analysis of anatomy-driven visibility limitations. Collectively, these properties support robust completeness monitoring and improved stability in downstream spinal curvature estimation (UCA).

Quantitatively, the proposed VPM-based framework achieved a mean vertebra-level completeness score of 0.91 using vertebra-centric 17-class detection, with a scan-level completeness score of 0.74 across T1–L5. A statistically significant and anatomically meaningful blind spot was identified at T7 in the per-vertebra analysis (accuracy = 0.729, *p* = 0.043), highlighting visibility challenges that are not captured by conventional detection metrics such as mAP.

### 6.2. Future Work

Future work will focus on (i) learnable acquisition guidance that recommends probe adjustments given VPM feedback, (ii) temporal consistency modelling across cine frames to improve robustness under motion, and (iii) integration of VPM-driven completeness priors with generative reconstruction to recover persistently missing vertebrae and further stabilise downstream measurements. In our approach, the VPM is deliberately constructed explicitly as a post-object-detection anatomical reasoning step to maintain simplicity, interpretability, and broad applicability to existing object-detection pipelines. A future direction for this work would be to enable learnable formulations of anatomical completeness. Visibility and ordering constraints could be applied directly during model training, encouraging completeness via differentiable completeness objectives or sequence-level regularisation. While such an approach falls outside the scope of this work, which seeks to provide an explicit and model agnostic step for completeness analysis, we view learnable notions of anatomical constraints as a promising direction for enforcing anatomical correctness and improving robustness in ultrasound-based scoliosis assessment.

## Figures and Tables

**Table 1:** Comparison across four YOLO configurations.

Scenario	Dataset	Number of Classes	Validation Images (n)	Validation Instances (GT Boxes)	Precision (Box)	Recall (Box)	mAP@0.50	mAP@0.50–0.95	Note
YOLO17-Full class	Cleaned ROI	17	472	472	0.174	0.567	0.254	0.176	Multi-class full-spine detection is unstable under anatomical clutter; weak backbone for downstream UCA estimation without additional reasoning.
YOLO1-Full class	Cleaned ROI	1	472	110	0.374	0.618	0.453	0.360	Detects vertebra presence more reliably than YOLO17-Full, but still below crop-based configurations.
YOLO17-Crop class	Vertebra crops	17	729	729	0.886	0.868	0.929	0.929	Stable vertebra-level identification enabled by one-to-one crop design; supports VPM completeness analysis.
YOLO1-Crop class	Vertebra crops	1	729	729	1.000	1.000	0.995	0.995	Near upper-bound localisation without class ambiguity; used as a reference for VPM construction.

**Table 2:** VPM-based completeness metrics for the four YOLO configurations.

Model	Overall Completeness Accuracy	Per-Scan Completeness Rate	Mean Missing-Vertebra Frequency
YOLO17-Full	0.41	0.09	0.58
YOLO1-Full	0.63	0.21	0.37
YOLO17-Crop	0.91	0.74	0.09
YOLO1-Crop	0.98	0.94	0.01

**Table 3:** Per-vertebra VPM accuracy for YOLO17-Crop on the validation set (n=729 images).

Vertebra	*N* Vertebra–Image Pairs	Correct VPM Matches	VPM Accuracy
T1	27	25	0.926
T2	40	35	0.875
T3	43	36	0.837
T4	28	22	0.786
T5	45	35	0.778
T6	25	22	0.880
T7	48	35	0.729
T8	23	22	0.913
T9	41	37	0.902
T10	24	18	0.750
T11	42	36	0.857
T12	30	27	0.900
L1	66	55	0.833
L2	58	45	0.776
L3	62	55	0.887
L4	61	52	0.853
L5	66	51	0.773

## Data Availability

The data presented in this study are not publicly available due to ethical and privacy restrictions.

## Abbreviations

UCA	Ultrasound Curve Angle
VPM	Vertebra Presence Matrix
